# Novel Insight into the Volatile Profile and Antioxidant Properties of *Crocus sativus* L. Flowers

**DOI:** 10.3390/antiox11091650

**Published:** 2022-08-25

**Authors:** Débora Cerdá-Bernad, Jesús Clemente-Villalba, Estefanía Valero-Cases, Joaquín-Julián Pastor, María-José Frutos

**Affiliations:** 1 Research Group on Quality and Safety, Agro-Food Technology Department, CIAGRO-UMH, Centro de Investigación e Innovación Agroalimentaria y Agroambiental, Miguel Hernández University, 03312 Orihuela, Spain; 2 Engineering Department, CIAGRO-UMH, Centro de Investigación e Innovación Agroalimentaria y Agroambiental, Miguel Hernández University, 03312 Orihuela, Spain

**Keywords:** saffron, floral bio-residues, volatile compounds, polyphenols, flavonoids, anthocyanins, apocarotenoids, sustainability, functional food, high added-value ingredients

## Abstract

The current production system of saffron spice generates hundreds of tons of waste. Thus, the aim of this study was to value both saffron and its floral by-products as a source of natural bioactive extracts, studying the in vitro antioxidant capacity, the composition of the volatile fraction by GC-MS/MS, and the determination of crocetins esters by HPLC-PDA. Saffron stigmas and floral by-products showed a high content of polyphenols and different antioxidant properties. Floral bio-residues (tepals, stamens, and styles) presented a high concentration of anthocyanins, and stigmas had high levels of flavonoids, β-carotene, and total crocins. In stigmas, 25 different volatile components were found, with safranal the most relevant. Floral by-products volatile composition consisted of 55 compounds with varying amounts depending on the drying treatment; all the samples presented acetic acid, 2(5H)-furanone, and phenylethyl alcohol. Therefore, saffron stigmas and flower by-products represent a sustainable source of bioactive ingredients for innovative healthy food formulations.

## 1. Introduction

Saffron (*Crocus sativus* L.), a traditional Mediterranean plant, is a perennial herb that belongs to the Iridaceae family. It is employed as a spice, natural colorant in food, and a flavoring agent. This spice is obtained from the flower of *C. sativus*, which is composed of three golden yellow stamens, six purple tepals, and one red pistil that culminates with three red branched stigmas, whose length exceeds that of the tepals, which, when dried up, constitute the saffron spice [1]. Only flower stigmas are used for saffron production, while tepals and stamens are simply discarded. For the production of 1 kg of saffron, around 350 kg of tepals are generated as a by-product. Thus, the current production system is not sustainable since it generates hundreds of tons of waste, with a high environmental impact [2].

In addition to its organoleptic properties, saffron is occasionally used as a textile dye, in perfumes, and in medicine due to its therapeutic properties since it has been considered for centuries as a medicinal plant in many cultures [3]. The therapeutic activity of saffron is mainly due to its valuable bioactive compounds (carotenoids, terpenes, and flavonoids). The main chemical components are crocin, crocetin and safranal. Crocetin, the principal apocarotenoid in saffron, is the precursor of crocin, and the most abundant compound is *trans*-crocetin di(β-D-gentiobiosyl) ester. The volatile compounds, including terpenoids, along with phenolic compounds that are widely distributed in plants, are responsible for their sensory properties. In saffron, 3-Cyclohexadiene-1-carboxaldehyde, 2,6,6-trimethyl-, known as safranal, is the major volatile compound contributing to saffron aroma [4]. 

One of the greatest interests of these bioactive compounds is due to their high antioxidant capacity and free radical scavenging activity. The generation of reactive oxygen species is a normal process in cells, but the uncontrolled generation and concomitant increase in reactive oxygen species (ROS) level in the body results in “oxidative stress”, which is considered to be the main cause of various diseases [5]. The antioxidant substances play a role in protecting biological systems against the effects of oxidative processes on macromolecules. Many of those substances are plant-derived natural molecules, such as carotenoids or polyphenolic compounds, that could contribute to preventing and fighting against several diseases in which ROS are involved [6].

Currently, some studies have reported that tepals, considered waste, contain valuable bioactive compounds such as flavonoids and polyphenols with potential antioxidant activity [7]. Therefore, saffron floral by-products could be natural antioxidant sources to be used in food, increasing the resource efficiency and the competitiveness of this sector. This waste raw material could be valorized through the development of innovative high added-value food ingredients, and this could potentially increase saffron demand on the market. 

The main objective of this study was to value both saffron and its floral by-products through their use as a source of bioactive extracts, studying the in vitro antioxidant capacity, the metal chelating activity, the composition of volatile compounds by GC-MS/MS, and the determination of crocetins esters and total crocins by HPLC in saffron and its floral by-products. As far as we know, no research has been published to date that delves into the composition of the volatile fraction in saffron floral by-products and into the changes in the volatile profile depending on the drying treatment used. Moreover, the results of this research would support the production of saffron as a more sustainable and profitable agronomic resource.

## 2. Materials and Methods

### 2.1. Chemicals

Methanol, ethanol, n-hexane, acetone, and acetonitrile solutions were HPLC grade (J.T. Baker, Madrid, Spain), and hydrochloric acid 37% was obtained from Panreac (Barcelona, Spain).

Trolox, 2,2-diphenyl-1-picrylhydrazyl (DPPH), 2,2′-azinobis (3-ethylbenzothiazoline-6-sulfonic acid) (ABTS), potassium persulfate (K_2_S_2_O_8_), 2,4,6-tris(2-pyridyl)-s-triazine (TPTZ), iron(III) chloride solution (FeCl_3_), iron(II) chloride solution (FeCl_2_), sodium acetate trihydrate (CH_3_COONa·3H_2_O), gallic acid analytical standard (≥98.0%), catechin analytical standard (≥99.0%), sodium nitrate (NaNO_2_), aluminum chloride (AlCl_3_), sodium hydroxide (NaOH), delphinidin chloride analytical standard (≥95.0%), sodium bisulfite (NaHSO_3_), ferrozine iron reagent, isoamyl acetate (≥95%), *trans*-crocetin di(β-D-gentiobiosyl) ester and *trans*-crocetin (β-Dglucosyl)-(β-D-gentiobiosyl) ester standards (≥95%, HPLC grade) were purchased from Sigma Aldrich (St. Louis, MO, USA).

Folin–Ciocalteu reagent, sodium bicarbonate (Na_2_CO_3_), potassium dihydrogen phosphate (KH_2_PO_4_), and dipotassium hydrogen phosphate (K_2_HPO_4_) were purchased from Merck Millipore (Darmstadt, Germany). A commercial alkane standard mixture (C_6_-C_20_) for Gas Chromatography-Mass Spectrometry compounds identification was also purchased from Merck Millipore (Darmstadt, Germany).

For all the experiments, ultrapure Milli-Q water (Millipore Corp., Bedford, MA, USA) was used.

### 2.2. Plant Material

Saffron floral by-products (SFL1, SFL2) were obtained from different producers in Castilla-La Mancha region (Spain) during the 2020 harvest season and cultivated following the requirements established by the Protected Designation of Origin “La Mancha Saffron” according to DOCM [8]. The flowers were composed of all the parts of saffron flowers (tepals, stamens, and styles), except for the stigmas that were detached manually from the rest of the flower after being harvested, following the traditional procedure.

All fresh flowers were frozen in liquid nitrogen and stored at −80 °C until freeze-dried in a freeze-dryer Christ Alpha 2–4 (B. Braun Biotech International, Melsungen; Germany) for 48 h to constant weight (initial temperature −25 ± 2 °C and pressure 0.220 mbar). Then, they were crushed and sieved through a 500 μm mesh size and stored at −20 °C until further analysis. For the volatile composition analysis, in addition to fresh and freeze-dried samples, oven-dried samples were studied, which were dried for 24 h at 60 °C in an air oven.

Saffron stigmas were supplied dehydrated by the Spanish company Verdú Cantó Saffron Spain, S.L and were from Spanish (Castilla-La Mancha region), Greek (Kozani area), and Iranian cultivations (Torbat zone). The moisture of all samples was lower than 11%, and saffron threads were crushed and sieved through a 500 μm mesh size and stored at 4 °C until further analysis.

### 2.3. Extraction of Bioactive Compounds

#### 2.3.1. Polyphenolic Compounds

The extractions were prepared using a methanol solution and a sample/methanol ratio 1:20 (*w*/*v*) for the freeze-dried flowers and a ratio 1:50 (*w*/*v*) for saffron stigmas. The extracts were shaken for 1 h in the dark at 400 rpm with a magnetic stirrer (Ovan, mod. MultiMix Heat D-MMH30E) and then sonicated for 15 min and centrifuged at 10,000× *g* for 10 min at 4 °C. Then, the supernatants were filtered (0.45 µm PTFE filter, Millipore, Madrid, Spain) and stored at −20 °C. All extractions were performed in triplicate.

#### 2.3.2. Beta-Carotene

The extractions were prepared using n-hexane:acetone:ethanol (50:25:25, *v*/*v*/*v*) solution and a sample/solvent ratio 1:20 (*w*/*v*) for the freeze-dried flowers and a ratio 1:50 (*w*/*v*) for saffron stigmas. The extracts were shaken for 10 min at 400 rpm with a magnetic stirrer (Ovan, mod. MultiMix Heat D-MMH30E), keeping them in ice, and then centrifugated at 14,460× *g* for 20 min at 4 °C. Then, the supernatants were filtered (0.45 µm PTFE filter, Millipore, Madrid, Spain) and stored at −20 °C. All extractions were performed in triplicate.

#### 2.3.3. Anthocyanins

The extractions were prepared using 50% ethanol (0.1% HCl) solution and a sample/solvent ratio 1:20 (*w*/*v*) for the freeze-dried flowers. The extracts were sonicated for 30 min and centrifugated at 10,000× *g* for 15 min. Supernatants were filtered (0.45 µm PTFE filter, Millipore, Madrid, Spain). All extractions were performed in triplicate.

### 2.4. In Vitro Antioxidant Properties

For the study of the antioxidant properties, the extracts obtained from polyphenolic compounds extraction (Section 2.3.1.) were used.

#### 2.4.1. 2,2-Diphenyl-1-picrylhydrazyl (DPPH) Free Radical Scavenging Method

The free radical scavenging activity was determined using DPPH method, following the methodology from Brand-Williams et al. [9], with some modifications. The DPPH radical was prepared dissolving 0.0035 g with 10 mL of methanol. The mixture was shaken and kept in dark for 30 min. The absorbance decrease was measured at 515 nm (UV/Vis Spectrophotometer T80; PG Instruments Limited, UK). Trolox (10 mM) was used as a reference standard at different concentrations (0.50–4.00 mmol/L). The results were expressed as mmol of Trolox Equivalents (TE) per 100 g dw (dry weight) of sample. 

#### 2.4.2. 2,2′-Azinobis (3-ethylbenzothiazoline-6-sulfonic acid) (ABTS) Free Radical Scavenging Method

The ABTS cation radical method for measuring the antioxidant capacity was performed by adapting the methodology from Re et al. [10]. ABTS radical was prepared mixing ABTS (7 mM) with K_2_S_2_O_8_ (2.45 mM), and they reacted for 16 h in dark at room temperature. Subsequently, the solution was diluted with ultrapure water until its absorbance was adjusted to 0.70 ± 0.02 at 734 nm. Trolox (10 mM) was used as a reference standard in different concentrations (0.20–3.00 mmol/L). The results were expressed as mmol of TE per 100 g dw of sample. 

#### 2.4.3. Ferric Reducing Antioxidant Power (FRAP) Method

The FRAP method adopted from Benzie and Strain [11] was used. Briefly, the FRAP reagent was prepared fresh daily by mixing 300 mmol/L acetate buffer (pH 3.6), 10 mmol/L TPTZ solution in 40 mmol/L HCl, and 20 mmol/L FeCl_3_·6H_2_O solution in a volume ratio of 10:1:1, respectively. The absorbance was measured at 593 nm. Trolox (10 mM) was used as standard solution in the range of 0.01−5.00 mmol/L. The results were expressed as mmol of TE per 100 g dw of sample.

### 2.5. Bioactive Compounds Content

#### 2.5.1. Total Polyphenols Content (TPC)

The total polyphenols were determined using the Folin–Ciocalteu methodology following the method described by Singleton et al. [12]. The Folin–Ciocalteu reagent was mixed with ultrapure water 1:10 (*v*/*v*). Gallic acid (1 mM) was used as a reference standard in the range of 0.00–4.72 mg/L. For the assays, 100 μL of the different extracts were mixed with 400 μL of phosphate buffer (50 mM) at pH 7.8, and 2.5 mL of Folin–Ciocalteu reagent was added. After 2 min, 2 mL of Na_2_CO_3_ (75 g/L) were added and kept at 50 °C for 10 min. The absorbance was measured at 760 nm in a spectrophotometer (UV/Vis), and the results were expressed as mg Gallic Acid Equivalents (GAE) per g dw of sample.

#### 2.5.2. Total Flavonoids Content (TFC)

The total flavonoid content was determined as described by Çam and Hışıl [13]. Catechin was used as standard; in order to calculate the flavonoid content, a calibration curve ranging from 20–100 mg/L was prepared. The absorbance was measured at 510 nm, and the results were expressed in mg of Catechin Equivalents (CE) per g dw of sample. 

#### 2.5.3. Total Anthocyanins Content (TAC)

The total anthocyanin content was determined by the method from Figueira et al. [14] with some modifications. Delphinidin was used as an external standard, and the total anthocyanin content was calculated using a calibration curve (10–150 mg/L). The final absorbance was determined by the difference between the measured reference absorbance and the measured sample absorbance. The absorbance was measured at 520 nm, and the results were expressed in mg of Delphinidin Equivalents (mg DE) per g dw of sample.

#### 2.5.4. Total Beta-Carotene Content

The change in absorbance was measured using a UV-Vis spectrophotometer at 450 nm. Carotene content was determined using the Beer-Lambert law. The carotene concentration was calculated using the extinction coefficient of beta-carotene in hexane (2.505 M^−1^cm^−1^). The results were expressed as mg of beta-carotene per 100 g dw of sample (%).

### 2.6. Iron (II) Chelating Activity

For the metal chelating activity, the extractions were performed in the same procedure previously explained for the extraction of polyphenolic compounds but using ultrapure water as extracting agent.

The chelation of iron (II) ions was performed as described by Carter [15]. Briefly, a final concentration of 0.02 g/mL of extract were used and mixed with 100 µL of 2.0 mM aqueous FeCl_2_ and 900 µL methanol. After an incubation of 5 min, the reaction was initiated adding 400 µL of 5.0 mM ferrozine. Then, after a 10 min equilibrium period, the absorbance was measured at 562 nm. The iron chelation activities were calculated from the absorbance of the control (*Ac*) and of the sample (*As*) using the following equation and expressed as % inhibition:

(1) $$\%\;Inhibition=\frac{A_{c}-A_{s\;}}{A_{c}}\cdot 100$$

### 2.7. Color

The color was measured with a Minolta CR-300 Chroma Meter (Japan) colorimeter, using L*, a*, b* scale (CIELAB system). The results were expressed as luminosity L*, a* (greenness/redness), b* (blueness/yellowness), Hue angle (*h*) and Chroma (C*), which were calculated according to the following equations, respectively:

(2) $$h=\left(\arctan\left(\frac{b^{*}}{a^{*}}\;\right)\right)\times \frac{180}{\pi }$$

(3) $$C^{*}={\left(a^{*2}+b^{*2}\right)}^{1/2}$$

### 2.8. Determination of Crocetin Esters by HPLC-PDA

#### 2.8.1. Extract Preparation

The aqueous extracts of saffron stigmas and flowers were prepared according to ISO 3632 [16]. Briefly, 0.5 g of sample was mixed with 900 mL of ultrapure water in a 1 L volumetric flask. The solutions were shaken for 1 h in the dark at 1000 rpm on a magnetic stirrer. The flask was filled to 1 L and homogenized through agitation. Then, 20 mL of solution were transferred to a 200 mL volumetric flask which was filled with ultrapure water. The extractions were filtered (0.45 µm PTFE filter, Millipore, Madrid, Spain) and transferred into a vial for HPLC analysis. All extractions were performed in triplicate.

#### 2.8.2. HPLC-PDA

The identification and quantification of crocetin esters were carried out by high-performance liquid chromatography (HPLC) using HPLC Altus^TM^ 10 PerkinElmer (Waltham, MA, USA) equipped with a C18 KromaPhase column (150 × 4.6 mm inner diameter, 3.5 μm) (Scharlab, Barcelona, Spain) that was equilibrated at 30 °C. The analysis were carried out as described by Valle García-Rodríguez et al. [17] with some modifications. The eluents used were acetonitrile (A) and water (B), with a proportion of 20% A and 80% B. The flow rate was 0.8 mL/min, and the injection volume of saffron extracts was 25 μL. Crocetin esters were measured at 440 nm in UV-VIS (UV-6300PC double beam spectrophotometer) with the Photodiode Array (PDA) Detector (PerkinElmer, Waltham, MA, USA).

The identifications of crocetin esters, such as *trans*-crocetin di(β-D-gentiobiosyl) ester (*trans*-4-GG), and *trans*-crocetin (β-D-glucosyl)-(β-D-gentiobiosyl) ester (*trans*-3-Gg), were determined using the UV−VIS spectrum, and the retention time was carried out by the HPLC−PDA method at 440 nm. Their quantification was performed through calibration curves with the standards, y = 0.0075x − 0.0080 (R value = 0.99) for *trans*-4-GG in the range from 0.8 to 50 mg/L, and y = 0.0071x− 0.0047 (R value = 0.99) for *trans*-3-Gg concentration in the range from 0.8 to 25 mg/L.

### 2.9. Volatile Composition

The volatile composition was determined using headspace solid phase micro-extraction (HS-SPME). After several preliminary tests to optimize the extraction system, accurately amounts between 20 and 40 mg of saffron stigmas and between 120 and 140 mg of saffron flowers were weighted and added into a 40 mL vial with polypropylene caps and PTFE/silicone septa, and isoamyl acetate (1000 mg/L, internal standard for semi-quantification of compounds). Fresh, freeze-dried, and air oven-dried samples of saffron flowers were used in the analyses. Then, the vial was placed in an AOC-6000 Plus autosampler (Shimadzu Corporation, Kyoto, Japan), and after 5 min of equilibration time, a 50/30 μm DVB/CAR/PDMS fiber (1 cm) was exposed to the sample headspace for 45 min at 40 °C (with agitation, 250 rpm). 

The separation and identification of compounds were performed by GC2030 (Shimadzu Scientific Instruments, Inc., Columbia, MD, USA) in a Sapiens X5MS column (Teknokroma, Barcelona, Spain), 30 m × 0.25 mm i.d., 0.25 μm f.t., and coupled with a mass spectrometer detector (TQ8040 NX triple quadrupole mass spectrometer; Shimadzu Scientific Instruments, Inc., Columbia, MD, USA). Only the single quadrupole acquisition mode was used on the TQ8040 NX (Q3 Scan; scan speed 5000 amu/s; mass range 40–400 *m*/*z*; event time 0.100 s). The oven temperature program was as follows: (i) initial temperature of 35 °C and hold 5 min; (ii), increment of 5 °C/min up to 150 °C/min, and hold 1 min; (iii) increment of 10 °C/min up to 280 °C and hold for 5 min. Helium column head pressure was 47.6 kPa (constant linear velocity mode of 36 cm/s). Injector, ion source, and interface were at 250, 230, and 280 °C, respectively. Helium was used as gas carrier, column flow 1 mL/min, with split ratio 1:50 and purge flow of 6 mL/min.

Retention indexes of a commercial alkane standard mixture were used to identify the compounds, as well as the National Institute of Standards and Technology (NIST) 17 Mass Spectral and Retention Index Libraries. The identification was considered tentative when it was based only on mass spectral data, and only compounds with spectra similarity > 90% were considered correct hits. Linear retention similarity filter was set at ±10 units. This volatile compound extraction method has been previously used for the analysis of different food matrices, according to Clemente-Villalba et al. [18]. 

### 2.10. Statistical Analysis

All determinations were performed in triplicate. Results were expressed as the mean ± standard deviation. The mean comparisons were carried out using an analysis of variance (ANOVA) and by the Tukey multiple range test using SPSS version 21.0 software package (SPSS Inc., Chicago, IL, USA). The significant differences were established as *p* < 0.05.

## 3. Results and Discussion

### 3.1. Antioxidant Properties, Bioactive Content and Iron (II) Chelating Activity

Polyphenols, which are plant-derived natural molecules found as secondary metabolites, present important biological activities, with their antioxidant capacity one of the most important properties for physiological function. The total polyphenols and flavonoid content, as well as the antioxidant effects, were studied in saffron and its floral by-products extracts. 

The results of the antioxidant activity and bioactive content are shown in Table 1. Saffron floral by-products and saffron stigmas presented a high concentration of total polyphenols, between 32–36 mg GAE/g dw, except for Spanish saffron, which had a significant highest amount (44.80 ± 2.30 mg GAE/g dw). Regarding the total flavonoid content, saffron stigmas from Spain, Iran, and Greece showed high levels of flavonoids in the range of 15–18 mg CE/g dw. However, saffron flowers had a lower concentration of total flavonoids (4–5 mg CE/g dw) than saffron stigmas. 

These results obtained for saffron flowers were in accordance with the ones described by Sun et al. [19], reporting values about 30 mg/g for TPC and values lower than 10 mg/g for TFC in saffron tepal methanol extracts from China. Moreover, the values obtained for saffron stigmas were higher than those reported in the study of Karimi et al. [6], in which saffron stigmas methanol extracts from Iran presented 6.5 ± 0.02 mg GAE/g dw for TPC and 5.8 ± 0.12 mg rutin equivalents/g dw for TFC. 

Within the group of flavonoids, anthocyanins are one class of pigments that also present antioxidant properties [20]. Saffron flowers without stigmas, SFL1 and SFL2, contained high levels of anthocyanins, showing SFL2 a significantly higher concentration than SFL1. Serrano-Díaz et al. [21] also found the highest anthocyanin content in saffron tepals from Spain and were also detected in whole flowers and floral bio-residues. 

Regarding carotenoids, they are organic pigments from the group of isoprenoids that are found naturally in plants and exert several beneficial functions in the body due to their antioxidant properties, among others. Based on their structures, carotenes are one of the main subclasses of carotenoids, with β-carotene and α-carotene the two major types. Beta-carotene was found to be the precursor of crocins. Crocins, which are glycosyl esters of crocetin, are the main chemical components of saffron, responsible for many of its pharmacological and biomedical properties. The synthesis of these apocarotenoids involves several reactions, including the cleavage of beta-carotene and zeaxanthin, oxidation and glycosylation steps [22].

Saffron stigmas showed a high proportion of total beta-carotene being higher than 70%. Greek saffron presented the highest concentration (90.82 ± 8.37%) and Iranian saffron the lowest, having 20% less total beta-carotene than Greek saffron (71.06 ± 6.47%) (Table 1). With respect to the crocetin esters content, the *trans* isomers, which are the majority, were studied. *Trans*-4-GG crocin was found in higher concentrations than *trans*-3-Gg-crocin in all saffron samples. Spanish stigmas presented the significant highest proportion of total crocins (17.04 ± 0.18%) and of *trans*-4-GG crocin (11.91 ± 0.13%) (Table 2). The same tendency for beta-carotene content was shown by Iranian stigmas, having the lowest proportion of total crocins (13.19 ± 0.78%) and of *trans*-4-GG crocin (9.22 ± 0.30%) (Table 2).

These results were in accordance with those obtained by Valle García-Rodríguez et al. [17], that have reported the quantification of these two crocins (*trans*-4-GG and *trans*-3-Gg) in saffron from Italy, Iran, Greece, and Spain by HPLC. Furthermore, the values of Spanish saffron were consistent with the content of crocetin esters reported by Moratalla-López et al. [23], in which these compounds represented 16–28% of the saffron composition. Thus, the level of apocarotenoids may vary because of different geographical origins, processing, and storage conditions.

Saffron flowers presented low amounts of total beta-carotene (28–39%) that are mainly located in the yellow stamens. Nevertheless, beta-carotene was reported in higher quantity in other Crocus species such as *C. ancyrensis* also due to the yellow color of the tepals [22] (Table 1). Moreover, in saffron floral by-products, crocins were only detected in SFL1 but in very low amounts (0.042%) due to the presence of small fragments of stigmas remaining after their detachment (Table 2). However, Rubio Moraga et al. [22] reported that crocins in *C. sativus* saffron floral by-products from Spain were not detected. 

DPPH, ABTS, and FRAP assays results revealed the different antioxidant activities of saffron and its floral by-products (Table 1). DPPH and ABTS tests evaluate the in vitro antiradical activity, and FRAP assay the reducing potential of the extracts. Saffron stigmas presented stronger antioxidant capacity than the floral by-products, which could be related to the higher amounts of total flavonoids and crocins content since previous studies have demonstrated the significant antioxidant activity of flavonoids and crocins [24]. Nevertheless, saffron flowers, SFL1 and SFL2, showed a good antioxidant ability (by DPPH, ABTS, and FRAP) that could be related to their high polyphenols content. These results are similar to other studies that reported the antioxidant capacity of Indian and Iranian saffron stigmas [6,25], the potential antioxidant activity of commercial saffron powder from the large-scale Italian market [26], and also to that of Sun et al. [19] in which it is revealed the strong antioxidant ability of saffron tepals from China. 

**Figure 1:**
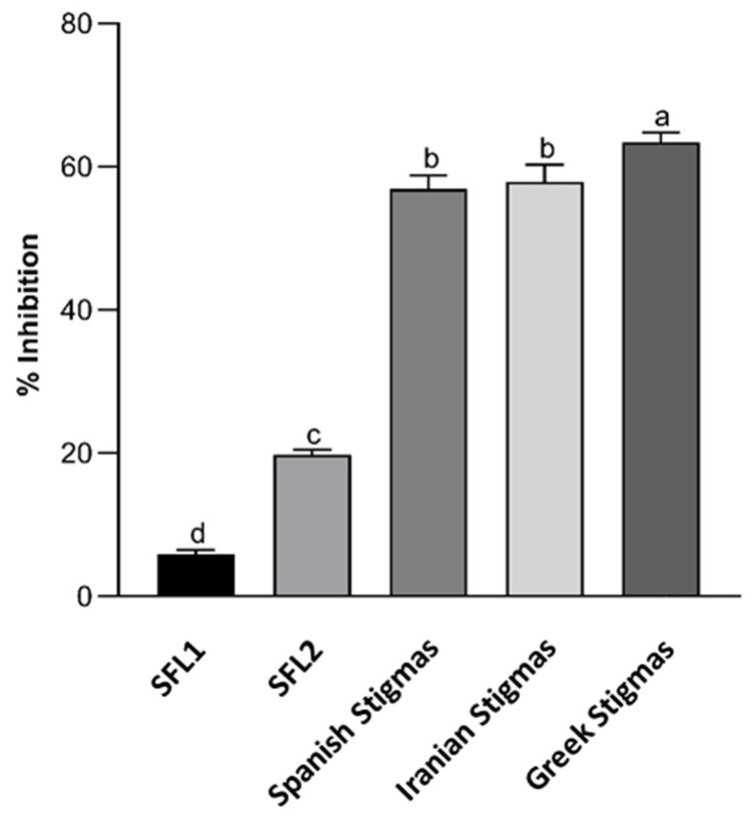
Iron (II) chelating activity of saffron floral-by products and saffron stigmas. The error bars represent the standard deviation and the different lowercase letters indicate statistically significant differences at *p* ≤ 0.05 for each sample (*n* = 3). SFL1, SFL2: Saffron floral by-products from two different producers.

Regarding the ability of the saffron extracts to chelate iron (II), the results are shown in Figure 1. Bivalent transition metal ions, such as iron, play an important role as catalysts of oxidative processes, participating in hydroxyl radical generation via the Fenton reaction [27]. Thus, excess metal ions could lead to the formation of free radicals generating high levels of oxidative stress, but these processes can be delayed by iron chelation. Apart from the ferric reducing power activity (FRAP) assay determination that indicated the reduction potential of Fe^3+^ to Fe^2+^, the iron (II) chelating activity of saffron stigmas and flowers was also studied. 

Saffron stigmas presented a good chelating activity, around 60% of inhibition, with the highest for the Greek saffron (Figure 1). This ability could be due to their flavonoid content since previous research demonstrated that these bioactive compounds have the capacity to neutralize reactive radicals and sequestrate metal ions, suppressing Fenton reactions [28]. Saffron flowers showed a low iron chelating activity (less than 20% of inhibition), with the capacity of SFL2 significantly higher than SFL1 (Figure 1). This fact might be related to the anthocyanins content of SFL2 (Table 1). These results were similar to those reported by Sánchez-Vioque et al. [29], indicating that saffron tepals from Spain had very low chelating activity. 

Therefore, the chelating activity of Fe^2+^ and antioxidant capacity that presented saffron and its floral by-products could prevent oxidative damage, protecting against oxidative stress. These abilities may be linked to their bioactive compounds content that were located differently on the flower; stigmas had carotenoids and flavonoids, and the rest of the flower presented anthocyanins and other polyphenolic compounds. 

### 3.2. Color

The color parameters of saffron and its floral by-products are shown in Table 3. The L* values were around 30 in all samples indicating a low luminosity and lightness. Regarding powdered saffron floral by-products, SFL1 and SFL2, positive a* values and negative b* values represented a color in the ranges of red and blue, respectively, which were characteristic of the violet color of tepals. It should be noted that the b* value of SFL2 (−4.77 ± 0.14) indicated a higher blue color intensity with respect to SFL1 (−1.63 ± 0.25). This fact may be mainly related to the high concentration of anthocyanins pigments found in SFL2, which render a blue, red, or purple color [30]. Moreover, *h* values (°) of SFL1 (334.50 ± 2.46) and SFL2 (313.00 ± 0.19) represented a tone in the blue/purple range, and the color was more saturated and intense in SFL2 than SFL1, represented by the chroma (C*) values (6.53 ± 0.18 and 3.78 ± 0.22, respectively). 

Saffron stigmas from Spain, Iran, and Greece showed positive a* and b* values, representing colors ranging from red to yellow, respectively, which are characteristic of saffron spice. The *h* values were around 26–28° which indicated a red-orange tone that was saturated and intense in all saffron samples (C* values around 10–12). This yellow to red color observed in the stigmas of *Crocus sativus* L. was due to the presence of a high amount of carotenoids, such as crocetin and crocins, which are responsible for the coloring power of the saffron spice exhibiting red, orange, and yellow colors [22].

The results showed that the color parameters variability within the flowers and stigmas of *Crocus sativus* L. is related to the number of bioactive compounds with coloring properties in the different parts of the plant. 

### 3.3. Volatile Composition

The evaluation of volatiles is an important aspect that contributes to the aroma of foods. Furthermore, volatile secondary metabolites also present important bioactive properties, such as antioxidant, antimicrobial, and anticancer activities [31]. 

The results of the volatile composition of saffron stigmas from Spain, Iran, and Greece are shown in Table 4.

In all saffron samples, a total of 25 different volatile compounds were identified and quantified, with the more representative chemical families, ketones (*n* = 8), terpenes (*n* = 8), aldehydes (*n* = 3), esters (*n* = 3), alcohols (*n* = 2) and acids (*n* = 1). Regarding the concentration of the different volatile compounds, there were significant differences between the samples, except for safranal (1,3-Cyclohexadiene-1-carboxaldehyde, 2,6,6-trimethyl-, V16) which was the compound found in a higher amount in all the samples regardless of the saffron origin (7443, 7656 and 7429 μg/g for Spanish, Iranian and Greek saffron, respectively). The concentrations of safranal were higher than those reported by other studies. Kanakis et al. [32] indicated maximum values of safranal in Greek saffron around 6879 μg/g, Culleré et al. [33] found 1365 ± 81.9 μg/g in Spanish saffron, and Jalali-Heravi et al. [34] showed values of 4356 μg/g for Iranian samples. These differences could be due to the post-harvest processing, such as the dehydration procedures since safranal is formed at elevated temperatures from picrocrocin (50–55 °C), but after this process, safranal could still be generated via *trans*-crocetin esters from other volatile compounds [4].

Other important volatile compounds presented in saffron stigmas belong to the family group of ketones (3) and alcohols (1), such as isophorone (V11); 1,4-cyclohexanedione, 2,2,6-trimethyl-(dihydrooxophorone, V14); 1-butanol, 3-methyl-(isoamyl alcohol, V2) and 2,6,6-trimethyl-2-cyclohexene-1,4-dione (4-keitoisophorone, V12). Regarding isophorone, isoamyl alcohol, and 4-keitoisophorone content, Greek saffron had a significant highest concentration of those compounds (1936 μg/g, 1018 μg/g, and 789 μg/g, respectively), with these values consistent with the results reported by Jalali-Heravi et al. [34] in Greek saffron. Other studies also reported that safranal, isophorone, 4-ketoisophorone, and dihydrooxophorone were the main volatiles found in saffron stigmas from central Italy [35]. Moreover, isophorone also has antimicrobial and antioxidant properties [36]. With respect to dihydrooxophorone, the significant highest content was found in Iranian saffron with a concentration of 848 μg/g; this value was higher than those obtained in other studies for Iranian saffron (591 μg/g) [37]. The formation of oxidized and reduced isophorone-related compounds might be produced through an enzymatic process or may occur via oxidation and decarboxylation of safranal and further chemical reactions of other compounds [4]. 

The volatile compounds identified in the saffron samples were comparable to those reported in previous research. Anastasaki et al. [38] reported that some of the major compounds in Spanish, Greek, Italian and Iranian samples were safranal, isophorone, dihydrooxophorone, but also 4-hydroxy-2,6,6-trimethyl-3-oxocyclohexa-1,4-diene-1-carboxaldehyde and 4-hydroxy-2,6,6-trimethylcyclohex-1-enecarbaldehyde (HTTC). Nevertheless, in the saffron samples studied, 4-hydroxy-2,6,6-trimethyl-3-oxocyclohexa-1,4-diene-1-carboxaldehyde (V22) and HTCC (V23) appeared in lower concentrations. HTCC is derived from picrocrocin, which is also the precursor of safranal and is formed under heat treatment by deglycosylation of picrocrocin or by hydrolysis [39]. Therefore, during the drying process, picrocrocin may produce safranal in high levels, thus producing lower concentrations of HTCC in saffron samples.

In summary, saffron from Greece had the highest concentration of total volatile compounds (around 13,067 μg/g) with respect to the Iranian and Spanish saffron samples (around 12,503 μg/g and 11,104 μg/g, respectively). These differences could be related to the geographical origin, which is considered an essential factor in the concentration of volatiles, among others, such as the harvest season, dehydration temperature and duration, and the storage time and conditions [39]. 

The aroma of saffron is mainly due to the contribution of aldehydes of saffron, such as safranal and its derivative 4-hydroxy-2,6,6-trimethyl-1-cyclohexene 1-carboxaldehyde, but other compounds could contribute as well, with the characteristic saffron aroma developed during the post-harvest treatment. With respect to the odor descriptors, the saffron aroma is especially herbal, spicy, sweet, fresh, woody, floral, and musty, among others (Table 4).

Regarding the flower by-products samples from Spain fresh and dried by two different methods (freeze-dried and air oven-dried), 55 volatile compounds were isolated, identified, and quantified (Table 5). The identification parameter of these compounds, namely Kovats index, retention time, chemical family, and odor descriptors, were indicated in Supplementary Table S1. 

The chemical families more representative found in all samples were: esters (*n* = 13), aldehydes (*n* = 12), and acids and alcohols (*n* = 5). All floral by-product samples presented acetic acid (V1), 2(5H)-furanone (V14), and phenylethyl alcohol (V31). SFL2 (air-oven dried) had the highest concentration of acetic acid (161.55 μg/g), followed by fresh SFL1 (145.21 μg/g). The production of this organic acid could occur by the conversion of some carbohydrates in the presence of oxygen.

SFL2 (air-oven dried) presented a significant highest level of 2(5H)-furanone (V14) (452 μg/g), while SFL1 (air-oven dried) showed the lowest concentration (4.11 μg/g). However, the generation of furanone, such as 2(5H)-furanone, may be due to several processes: via oxidation reactions, by microorganisms, or spontaneous formation via the Maillard reaction between sugars and amino acids during heating [40]. Thus, the content of this volatile compound could be related mainly to the sugar and amino acid composition of the fresh flowers SFL1 and SFL2, leading to different concentrations of 2(5H)-furanone in the air-oven dried flowers. Moreover, 2(5H)-furanone is authorized as a flavoring substance by the Joint FAO/WHO Expert Committee on Food Additives (JECFA) and is defined in its specification as a “rich winey meat-like aroma”. This fact could be interesting for the use of dried saffron flowers as food ingredients. This compound has also been studied as bactericidal [41].

Phenylethyl alcohol was present in the SFL2 (air-oven dried) sample in high concentrations (35.63 μg/g). However, minimal concentrations were found in the SFL1 (air-oven dried) sample (0.49 μg/g). This volatile was also found in other flower extracts (rose, hyacinth, geranium), presenting a rose-like odor and antimicrobial and antifungal properties, and it is widely used in foods and cosmetics [42].

It should be noted that safranal (V39) was only found in SFL2 (fresh and air-oven dried) but in very low amounts (0.11 and 7.53 μg/g, respectively). This could be due to the presence of traces of saffron stigmas in the floral by-products, with the amount of safranal higher in SFL2 (air-oven dried) since it is generated at the temperatures used (50–55 °C) for drying. Moreover, isophorone (V35) was also present in SFL2 (22.28 μg/g) which may be produced from safranal and/or from other precursors formed in the oven during the heating process. 

Other compounds that should be highlighted due to their high concentration were 3-hydroxy 2-butanone (acetoin, V4) and butanoic acid, 3-methyl (isovaleric acid, V9). These volatiles were found in fresh SFL1 at 299.42 μg/g and 177.52 μg/g, respectively. Acetoins, which exist widely in nature, are mainly found in higher plants that have the ability to synthesize them using different enzymes, but their biological mechanisms remain unclear; however previous studies have shown their antimicrobial actions [43]. Isovaleric acid is an isomer of valeric acid, which is a compound naturally present in plants as a metabolite that could be formed by the secondary metabolism of plants [44]. Moreover, these volatiles were only found in fresh samples, meaning that they may be degraded by heat.

In summary, some volatiles present in fresh flowers increased by air drying in the oven due to the thermal treatment, and new volatiles also appears in the air-dried flowers. Some volatile compounds (V3, V17, V26, V32, V44) were only found in SFL2 (air-oven dried) samples, which showed the highest concentration of total volatile compounds (833.49 μg/g). These results are justified by the generation of volatiles from other non-volatile precursors by heat processing. Thus, the post-harvest procedures, such as drying, are very important when considering the volatile quality in terms of the contribution of the volatile compounds that are generated during this processing step. 

## 4. Conclusions

The studied Spanish, Iranian, and Greek saffron stigmas and Spanish saffron floral by-products could have a great potential to develop new high-added-value ingredients due to their antioxidant properties and bioactive content. Floral by-products showed a good concentration of total anthocyanins, and saffron stigmas had a high concentration of total flavonoids, β-carotene, and crocetin esters. Furthermore, among the volatile composition, the saffron studied presented high levels of safranal, which contributes to the bioactivity and aroma of this spice. The floral by-products volatile composition from fresh to dried samples was highly influenced by the drying method, of which some may present biological activities, especially antioxidant and antimicrobial properties. Therefore, these results suggest that saffron and its floral by-products are natural sources of antioxidant compounds that could be used as sustainable, innovative ingredients to apply in food for the development of novel functional food products or for other human health applications.

## Figures and Tables

**Table 1:** Antioxidant capacity and bioactive compounds content in saffron floral by-products and stigmas ^1^.

	SFL1	SFL2	Spanish Stigmas	Iranian Stigmas	Greek Stigmas
DPPH ^2^	98.82 ± 4.79d	107.40 ± 1.95d	201.27 ± 7.71b	278.92 ± 8.37a	145.58 ± 21.98c
ABTS ^2^	50.99 ± 2.51c	48.41 ± 4.67c	110.88 ± 9.99b	142.22 ± 5.23a	124.95 ± 27.44ab
FRAP ^2^	1250 ± 88b	1181 ± 26b	3667 ± 319a	3471 ± 123a	3445 ± 274a
TPC ^3^	32.42 ± 6.90b	32.82 ± 2.23b	44.80 ± 2.30a	36.35 ± 3.47b	34.00 ± 3.22b
TFC ^4^	3.99 ± 0.79b	5.37 ± 0.19b	18.74 ± 1.22a	15.32 ± 1.73a	17.39 ± 4.14a
TAC ^5^	39.17 ± 2.98b	69.02 ± 4.34a	n.d.	n.d.	n.d.
TBC ^6^	28.39 ± 2.17c	39.59 ± 1.47c	77.51 ± 14.93ab	71.06 ± 6.47b	90.82 ± 8.37a

^1^ Means ± standard deviation in the same line followed by different lowercase letters indicate statistically significant differences at *p* ≤ 0.05 for each sample (*n* = 3). ^2^ The antioxidant capacity is expressed as mmol Trolox Equivalents (TE) per 100 g dw of sample. ^3^ The Total Polyphenols Content (TPC) are expressed as mg Gallic Acid Equivalents (GAE) per g dw of sample. ^4^ The Total Flavonoids Content (TFC) are expressed as mg of Catechin Equivalents (CE) per g dw of sample. ^5^ The Total Anthocyanin Content (TAC) were expressed in mg of Delphinidin Equivalents (mg DE) per g dw of sample. ^6^ The total bta-carotene is expressed as mg of beta-carotene per 100 g dw of sample (%). SFL1, SFL2: Saffron floral by-products from two different producers; n.d.: not determined.

**Table 2:** Crocins in saffron floral by-products and saffron stigmas ^1^.

	*trans*-4-GG (% *w*/*w*)	*trans*-3-Gg (% *w*/*w*)	Total Crocins (% *w*/*w*)
SFL1	0.009 ± 0.002d	0.034 ± 0.005b	0.042 ± 0.007c
SFL2	n.d.	n.d.	-
Spanish Stigmas	11.91 ± 0.13a	5.13 ± 0.05a	17.04 ± 0.18a
Iranian Stigmas	9.22 ± 0.30c	3.98 ± 0.48a	13.19 ± 0.78b
Greek Stigmas	10.74 ± 0.24b	3.93 ± 0.53a	14.67 ± 0.77b

^1^ Means ± standard deviation in the same column followed by different lowercase letters indicate statistically significant differences at *p* ≤ 0.05 for each sample (*n* = 3). SFL1, SFL2: Saffron floral by-products from two different producers; n.d.: not detected.

**Table 3:** Color parameters of the powdered saffron floral by-products (**A**) and saffron stigmas (**B**) ^1^.

(A)	SFL1	SFL2	(B)	Spanish Stigmas	Iranian Stigmas	Greek Stigmas
L*	32.29 ± 0.16a	30.94 ± 0.24b	L*	29.64 ± 0.12a	28.86 ± 0.18b	29.54 ± 0.15a
a*	3.41 ± 0.13b	4.45 ± 0.11a	a*	10.69 ± 0.01a	9.26 ± 0.17b	10.60 ± 0.10a
b*	−1.63 ± 0.25a	−4.77 ± 0.14b	b*	5.80 ± 0.13a	4.56 ± 0.09b	5.72 ± 0.16a
*h* (°)	334.50 ± 2.46a	313.00 ± 0.19b	*h* (°)	28.47 ± 0.34a	26.23 ± 0.25b	28.38 ± 0.41a
C*	3.78 ± 0.22b	6.53 ± 0.18a	C*	12.16 ± 0.15a	10.32 ± 0.19b	12.05 ± 0.25a

^1^ Means ± standard deviation in the same line followed by different lowercase letters indicate statistically significant differences at *p* ≤ 0.05 for each sample (*n* = 3). SFL1, SFL2: Saffron floral by-products from two different producers.

**Table 4:** Identification, concentration (μg/g) and descriptors of volatile compounds found in saffron stigmas ^1^.

Code	Compound	CF	RT	KI (EXP)	KI (LIT)	Spanish Stigmas (μg/g)	Iranian Stigmas (μg/g)	Greek Stigmas (μg/g)	Odor Descriptors ^2^
V1	Acetic acid	Organic acid	2.422	645	646	18.30c	40.40a	35.80b	Pungent, sour, vinegar
V2	1-Butanol, 3-methyl- (isoamyl alcohol)	Alcohol	5.445	744	750	311c	770b	1018a	Alcoholic, whiskey, fruity, banana
V3	Cyclopetanone	Ketone	6.750	774	767	2.01a	0.63c	1.65b	Minty
V4	2(5H)-Furanone	Ester	12,.27	903	913	173a	123b	63.50c	Buttery
V5	Hexyl acetate	Ester	16.070	1012	1012	3.62c	10.10a	9.36b	Sweet, green, fruity, banana
V6	3-Cyclohexen-1-one, 3,5,5-trimethyl- (beta isophorone)	Ketone	17.041	1040	1044	5.40c	7.60b	8.92c	Woody, sweet, camphoreous, musty
V7	Linalool	Terpene	19.026	1098	1098	5.50a	3.97b	5.26a	Floral, citrus, rose
V8	Nonanal	Aldehyde	19.159	1101	1101	7.40a	1.71c	6.08b	Waxy, aldehydic, citrus, fresh
V9	Cyclohexene, 1-methyl-4-(1-methylethylidene)- (Terpinolene)	Terpene	19.256	1105	1098	49.00b	49.20b	59.50a	Herbal, fresh, sweet, pine
V10	Phenylethyl Alcohol	Alcohol	19.389	1109	1110	22.80c	39.80b	110a	Floral, rose
V11	Isophorone	Ketone	19.687	1119	1118	1477b	1531b	1936a	Woody, sweet, camphoreous, musty
V12	2,6,6-Trimethyl-2-cyclohexene-1,4-dione (4-ketoisophorone)	Ketone	20.409	1142	1139	273c	690b	789a	Musty, woody, sweet
V13	2-Hydroxy-3,5,5-trimethyl-cyclohex-2-enone	Ketone	20.504	1145	1149	20.10b	39.70a	14.80c	Woody, dry nutty, tobacco
V14	1,4-Cyclohexanedione, 2,2,6-trimethyl- (Dihydrooxophorone)	Ketone	21.181	1166	1168	748c	848a	792c	Woody, musty, sweet
V15	Benzaldehyde, 2,4-dimethyl-	Aldehyde	21.693	1183	1180	17.30a	13.00b	12.90b	Naphthyl, cherry, almond, spice, vanilla
V16	1,3-Cyclohexadiene-1-carboxaldehyde, 2,6,6-trimethyl- (Safranal)	Terpene	22.191	1198	1197	7443a	7656a	7429a	Fresh, herbal, saffron, spicy
V17	2,4-Cycloheptadien-1-one, 2,6,6-trimethyl- (Eucarvone)	Terpene	22.817	1220	1222	54.00c	120b	127a	Minty
V18	Acetic acid, 2-phenylethyl ester	Ester	23.757	1252	1250	8.30c	31.60b	72.90a	Floral, honey, fruity, tropical
V19	4-Hydroxy-3,5,5-trimethylcyclohex-2-enone	Ketone	25.339	1307	1317	16.60c	54.10a	35.40b	Camphor
V20	Benzaldehyde, 2,4,6-trimethyl-	Aldehyde	25.563	1315	1323	4.40c	6.89a	5.01b	Naphthyl, cherry, almond, vanilla
V21	α-Cubebene	Terpene	27.321	1379	1372	4.00a	1.36c	3.20b	Herbal, waxy
V22	4-hydroxy-2,6,6-trimethyl-3-oxocyclohexa-1-ene-1-carbaldehyde	Ketone	27.429	1383	1396	289b	286b	311a	Citrus, vegetable
V23	4-Hydroxy-2,6,6-trimethylcyclohex-1-enecarbaldehyde (HTTC)	Terpene	28.380	1418	1431	140c	172b	211a	Tropical, saffron, herbal
V24	2-Butanone, 4-(2,6,6-trimethyl-1-cyclohexen-1-yl)-	Terpene	28.802	1434	1433	7.90a	3.80c	5.68b	Earthy, woody
V25	3-Buten-2-one, 4-(2,6,6-trimethyl-1-cyclohexen-1-yl)-	Terpene	30.048	1480	1486	3.30c	4.00b	4.43c	Woody, sweet, fruity, berry
	Total					11,104.03	12,503.86	13,067.39	

^1^ Means in the same line followed by different lowercase letters indicate statistically significant differences at *p* ≤ 0.05 for each sample (*n* = 3); CF: Chemical Family; RT: Retention Time; KI: Kovats Index; EXP: Experimental; LIT: Literature; ^2^ Commercial flavor descriptors. or online according to: Flavornet (http://www.flavornet.org/flavornet.html) (accessed on 2 July 2022); Bedoukian Research (http://www.bedoukian.com/) (accessed on 2 July 2022); Sigma Aldrich SAFC. Flavors and Fragrances (http://www.safcglobal.com/safc-supply-solutions/en-us/home/flavors-and-fragrances.html) (accessed on 2 July 2022) and The Good Scents Company (http://www.thegoodscentscompany.com/) (accessed on 2 July 2022); FAO/WHO Expert Committee on Food Additives (JECFA) (https://www.fao.org/food/food-safety-quality/scientific-advice/jecfa/en/) (accessed on 2 July 2022).

**Table 5:** Identification and concentration (μg/g) of volatile compounds found in saffron floral by-products ^1^.

Code	Compound	RT	SFL1 Fresh (μg/g)	SFL1 Freeze-Dried (μg/g)	SFL1 Air-Oven Dried (μg/g)	SFL2 Fresh (μg/g)	SFL2 Freeze-Dried (μg/g)	SFL2 Air-Oven Dried (μg/g)
V1	Acetic acid	2.441	145.21b	5.09cd	0.26d	8.20c	0.83d	161.55a
V2	Butanal, 3-methyl-(Isovaleraldehyde)	3.370	13.66b	n.d.	n.d.	n.d.	n.d.	28.30a
V3	Butanal, 2-methyl-	3.531	n.d.	n.d.	n.d.	n.d.	n.d.	50.93
V4	3-hydroxy-2-butanone (Acetoin)	4.531	299.42	n.d.	n.d.	n.d.	n.d.	n.d.
V5	1-Butanol, 2-methyl-	5.617	5.84	n.d.	n.d.	n.d.	n.d.	n.d.
V6	Propanoic acid, 2-methyl-	6.163	3.13	n.d.	n.d.	n.d.	n.d.	n.d.
V7	2,3-Butanediol	7.405	2.61	n.d.	n.d.	n.d.	n.d.	n.d.
V8	Hexanal	7.888	n.d.	1.52	n.d.	n.d.	n.d.	n.d.
V9	Butanoic acid, 3-methyl- (Isovaleric acid)	10.222	177.52	n.d.	n.d.	n.d.	n.d.	n.d.
V10	Butanoic acid, 2-methyl-	10.364	8.26	n.d.	n.d.	n.d.	n.d.	n.d.
V11	1,2-Propanediol, 2-acetate	11.489	2.51	n.d.	n.d.	n.d.	n.d.	n.d.
V12	Heptanal	11.941	n.d.	3.96	n.d.	n.d.	n.d.	n.d.
V13	4-Penten-1-yl acetate	11.962	n.d.	n.d.	3.67	n.d.	n.d.	n.d.
V14	2(5H)-Furanone	12.104	4.42b	38.01b	4.11b	14.64b	4.95b	452a
V15	Butyrolactone	12.127	n.d.	24.47	n.d.	n.d.	n.d.	n.d.
V16	Acetic acid, pentyl ester	12.449	3.40e	3.95d	11.12a	8.85b	5.81c	n.d.
V17	2-Furancarboxaldehyde, 5-methyl-	14.186	n.d.	n.d.	n.d.	n.d.	n.d.	6.92
V18	1-Butanol, 3-methyl-, propanoate	14.551	n.d.	0.56d	3.16a	2.20b	0.84c	n.d.
V19	Carbolic acid	14.816	7.60	n.d.	n.d.	n.d.	n.d.	n.d.
V20	Diisoamyl ether	15.746	n.d.	n.d.	1.13	n.d.	n.d.	n.d.
V21	Acetic acid, hexyl ester	16.080	n.d.	1.13c	4.22a	2.86b	0.90d	n.d.
V22	1-Hexanol, 2-ethyl-	16.653	n.d.	n.d.	n.d.	n.d.	13.38	n.d.
V23	D-Limonene	16.657	n.d.	n.d.	n.d.	0.59	n.d.	n.d.
V24	Benzeneacetaldehyde	17.099	n.d.	3.86c	n.d.	4.27b	1.11d	13.39a
V25	Butanoic acid, pentyl ester	17.596	n.d.	n.d.	0.65a	0.58b	n.d.	n.d.
V26	Ethanone, 1-(1H-pyrrol-2-yl)-	17.717	n.d.	n.d.	n.d.	n.d.	n.d.	25.28
V27	Benzaldehyde, 4-methyl-	18.448	n.d.	0.53	n.d.	n.d.	n.d.	n.d.
V28	2-Nonanone	18.734	3.44	n.d.	n.d.	n.d.	n.d.	n.d.
V29	Linalool	19.031	2.46a	n.d.	n.d.	n.d.	0.77b	n.d.
V30	Nonanal	19.193	n.d.	12.40b	0.62c	n.d.	0.94c	14.98a
V31	Phenylethyl Alcohol	19.404	7.37d	10.03c	0.49f	16.64b	3.89e	35.63a
V32	4H-Pyran-4-one, 2,3-dihydro-3,5-dihydroxy-6-methyl-(DDMP)	20.326	n.d.	n.d.	n.d.	n.d.	n.d.	62.83
V33	Acetic acid, 2-ethylhexyl ester	20.559	n.d.	n.d.	n.d.	0.52	n.d.	n.d.
V34	2(3H)-Furanone, dihydro-4-hydroxy-	20.609	n.d.	109.82a	n.d.	n.d.	9.01b	n.d.
V35	Isophorone	20.675	n.d.	n.d.	n.d.	n.d.	n.d.	22.28
V36	Cyclohexanone, 5-methyl-2-(1-methylethyl)-, trans- (trans-Menthone)	20.859	n.d.	n.d.	n.d.	n.d.	0.60	n.d.
V37	Acetic acid, phenylmethyl ester	21.000	n.d.	n.d.	n.d.	0.30b	0.84a	n.d.
V38	Cyclohexanol, 5-methyl-2-(1-methylethyl)-, (1α,2β,5α)-(Menthol)	21.529	n.d.	n.d.	n.d.	n.d.	0.98	n.d.
V39	1,3-Cyclohexadiene-1-carboxaldehyde, 2,6,6-trimethyl-(Safranal)	22.180	n.d.	n.d.	n.d.	0.11b	n.d.	7.53a
V40	Benzofuran, 2,3-dihydro- (Coumaran)	22.630	2.59	n.d.	n.d.	n.d.	n.d.	n.d.
V41	2-Dodecene, (Z)-	22.375	n.d.	n.d.	n.d.	n.d.	1.20	n.d.
V42	3-Dodecene, (Z)-	22.634	n.d.	n.d.	n.d.	n.d.	1.14	n.d.
V43	Benzaldehyde, 2,4-dimethyl-	22.659	n.d.	n.d.	0.32	n.d.	n.d.	n.d.
V44	2-Cyclohexen-1-one, 2-methyl-5-(1-methylethyl)-, (S)-	23.656	n.d.	n.d.	n.d.	n.d.	n.d.	14.98
V45	Benzene, 1,3-bis(1,1-dimethylethyl)-	23.668	n.d.	3.75a	0.47b	0.55b	n.d.	n.d.
V46	Acetic acid, 2-phenylethyl ester	23.773	9.96b	2.34d	n.d.	32.42a	3.72c	n.d.
V47	Benzeneacetaldehyde, α-ethylidene-	24.200	n.d.	n.d.	n.d.	0.97	n.d.	n.d.
V48	2-Propenal, 3-phenyl-	24.337	n.d.	n.d.	n.d.	0.50	n.d.	n.d.
V49	Dodecane, 4,6-dimethyl-	24.450	n.d.	n.d.	0.34c	0.67bc	0.92b	16.12a
V50	2-Undecanone	24.903	0.73	n.d.	n.d.	n.d.	n.d.	n.d.
V51	α-Cubebene	27.334	n.d.	n.d.	n.d.	1.00a	0.53b	n.d.
V52	Dodecanal	28.113	n.d.	n.d.	n.d.	n.d.	1.79	n.d.
V53	1-Dodecanol	29.900	n.d.	n.d.	n.d.	n.d.	4.63	n.d.
V54	Lauryl acetate	32.679	n.d.	n.d.	0.48	n.d.	n.d.	n.d.
V55	Hexadecanoic acid, methyl ester	37.034	0.93	n.d.	n.d.	n.d.	n.d.	n.d.
	Total		700.13	217.46	27.37	93.90	58.78	833.49

^1^ Means in the same line followed by different lowercase letters indicate statistically significant differences at *p* ≤ 0.05 for each sample (*n* = 3); RT: Retention Time; SFL1, SFL2: Saffron floral by-products from two different producers; n.d.: not detected.

## Supplementary Materials

The following supporting information can be downloaded at: https://www.mdpi.com/article/10.3390/antiox11091650/s1, Table S1: Identification, quantification and descriptors of volatile compounds found in saffron floral by-products.
