# Supplementary material for: Novel Insight into the Volatile Profile and Antioxidant Properties of Crocus sativus L. Flowers
Source: Antioxidants (Basel). 2022 Aug 25;11(9):1650. doi: 10.3390/antiox11091650 (PMC9495350; doi:10.3390/antiox11091650)
Supplement: Supplementary file 1 [file antioxidants-11-01650-s001.zip › antioxidants-1864173-supplementary.pdf]

Table S1. Identification, quantification and descriptors of volatile compounds found in saffron floral by-products.

Code	Compound	CF	RT	KI LIT	KI EXP	Odor Descriptors [1]
**V1**	Acetic acid	Acid	2.441	645	646	Pungent, sour, vinegar
**V2**	Butanal, 3-methyl- (Isovaleraldehyde)	Aldehyde	3.370	690	686	Chocolate, peach, fatty
**V3**	Butanal, 2-methyl-	Aldehyde	3.531	699	688	Cocoa, musty, coffee
**V4**	3-hydroxy-2-butanone (Acetoin)	Ketone	4.531	722	721	Buttery, dairy, fatty
**V5**	1-Butanol, 2-methyl-	Alcohol	5,617	748	748	Fusel, alcoholic
**V6**	Propanoic acid, 2-methyl-	Acid	6.163	760	757	Sour, cheesy, rancid
**V7**	2,3-Butanediol	Alcohol	7.405	789	786	Creamy, fruity, buttery
**V8**	Hexanal	Aldehyde	7.888	800	800	Green, fresh, fatty
**V9**	Butanoic acid, 3-methyl- (Isovaleric acid)	Acid	10.222	858	858	Cheesy, sour
**V10**	Butanoic acid, 2-methyl-	Acid	10.364	862	861	Cheesy, acidic
**V11**	1,2-Propanediol, 2-acetate	Ester	11.489	889	891	Fruity
**V12**	Heptanal	Aldehyde	11.941	894	893	Green, fresh, fatty
**V13**	4-Penten-1-yl acetate	Ester	11.962	895	890	Green, plastic, vegetable
**V14**	2(5H)-Furanone	Ester	12.104	906	913	Buttery
**V15**	Butyrolactone	Ester	12.127	906	908	Creamy, oily, fatty
**V16**	Acetic acid, pentyl ester	Ester	12.449	914	912	Fruity, banana, pear
**V17**	2-Furancarboxaldehyde, 5-methyl-	Aldehyde	14.186	960	961	Caramellic, sweet, coffee
**V18**	1-Butanol, 3-methyl-, propanoate	Acid	14.551	970	969	Fruity, tropical, banana
**V19**	Carbolic acid	Acid	14.816	977	976	Plastic
**V20**	Diisoamyl ether	Ether	15.746	1002	1002	Floral
**V21**	Acetic acid, hexyl ester	Ester	16.080	1012	1011	Fruity, green
**V22**	1-Hexanol, 2-ethyl-	Alcohol	16.653	1029	1029	Citrus, fresh, floral
**V23**	D-Limonene	Terpene	16.657	1029	1029	Citrus, fresh
**V24**	Benzeneacetaldehyde	Aldehyde	17.099	1042	1043	Green, sweet, floral
**V25**	Butanoic acid, pentyl ester	Ester	17.596	1056	1059	Fruity, banana, tropical
**V26**	Ethanone, 1-(1H-pyrrol-2-yl)-	Ketone	17.717	1060	1060	Musty, nut
**V27**	Benzaldehyde, 4-methyl-	Aldehyde	18.448	1081	1080	Fruity, cherry

**V28**	2-Nonanone	Ketone	18.734	1089	1089	Fresh, sweet, fruity
**V29**	Linalool	Terpene	19.031	1098	1098	Floral, citrus, rose
**V30**	Nonanal	Aldehyde	19.193	1103	1102	Waxy, aldehydic, citrus, fresh
**V31**	Phenylethyl Alcohol	Alcohol	19.404	1110	1110	Floral, rose
**V32**	4H-Pyran-4-one, 2,3-dihydro-3,5-dihydroxy-6-methyl- (DDMP)	Other	20.326	1139	1140	-
**V33**	Acetic acid, 2-ethylhexyl ester	Ester	20.559	1147	1157	Earthy, herbal
**V34**	2(3H)-Furanone, dihydro-4-hydroxy-	Ester	20.609	1148	1153	-
**V35**	Isophorone	Ketone	20.675	1150	1146	Woody, sweet, camphoreous, musty
**V36**	Cyclohexanone, 5-methyl-2-(1-methylethyl)-, trans- (trans-Menthone)	Cetone	20.859	1156	1152	Minty
**V37**	Acetic acid, phenylmethyl ester	Ester	21.000	1161	1161	Floral, jasmine
**V38**	Cyclohexanol, 5-methyl-2-(1-methylethyl)-, (1α,2β,5α)- (Menthol)	Cetone	21.529	1177	1171	Minty
**V39**	1,3-Cyclohexadiene-1-carboxaldehyde, 2,6,6-trimethyl- (Safranal)	Terpene	22.180	1198	1201	Fresh, herbal, saffron, spicy
**V40**	Benzofuran, 2,3-dihydro- (Coumaran)	Other	22.630	1213	1219	Sweet
**V41**	2-Dodecene, (Z)-	Alkene	22.375	1205	1213	-
**V42**	3-Dodecene, (Z)-	Alkene	22.634	1214	1222	-
**V43**	Benzaldehyde, 2,4-dimethyl-	Aldehyde	22.659	1214	1208	Naphthyl, sweet
**V44**	2-Cyclohexen-1-one, 2-methyl-5-(1-methylethyl)-, (S)-	Terpene	23.656	1249	1250	-
**V45**	Benzene, 1,3-bis(1,1-dimethylethyl)-	Other	23.668	1249	1249	-
**V46**	Acetic acid, 2-phenylethyl ester	Ester	23.773	1252	1254	Floral, honey, fruity, tropical
**V47**	Benzeneacetaldehyde, α-ethylidene-	Aldehyde	24.200	1267	1273	Green, floral, woody
**V48**	2-Propenal, 3-phenyl-	Aldehyde	24.337	1272	1278	Spicy, sweet
**V49**	Dodecane, 4,6-dimethyl-	Alkane	24.450	1276	1285	-
**V50**	2-Undecanone	Cetone	24.903	1291	1291	Fruity, waxy,fatty
**V51**	α-Cubebene	Terpene	27.334	1379	1380	Herbal, waxy
**V52**	Dodecanal	Aldehyde	28.113	1408	1409	Soapy, waxy
**V53**	1-Dodecanol	Alcohol	29.900	1475	1474	Waxy, earthy, soapy
**V54**	Lauryl acetate	Ester	32.679	1606	1605	Waxy, sweet
**V55**	Hexadecanoic acid, methyl ester	Ester	37.034	1921	1921	Waxy, oily, fatty

[1] Commercial flavor descriptors. or online according to: Flavornet (http://www.flavornet.org/flavornet.html); Bedoukian Research (http://www.bedoukian.com/); Sigma Aldrich SAFC. Flavors and Fragrances (http://www.safcglobal.com/safc-supply-solutions/en-us/home/flavors-and-fragrances.html) and The Good Scents Company (http://www.thegoodscentscompany.com/); FAO/WHO Expert Committee on Food Additives (JECFA)(https://www.fao.org/food/food-safety-quality/scientific-advice/jecfa/en/)
CF: Chemical Family; RT: Retention Time; KI: Kovats Index; EXP: Experimental; LIT: Literature.
